# Origin of the natural variation in the storage of dietary carotenoids in freshwater amphipod crustaceans

**DOI:** 10.1371/journal.pone.0231247

**Published:** 2020-04-15

**Authors:** Aurélie Babin, Sébastien Motreuil, Maria Teixeira, Alexandre Bauer, Thierry Rigaud, Jérôme Moreau, Yannick Moret

**Affiliations:** Equipe Ecologie Evolutive, UMR CNRS 6282 Biogéosciences, Université de Bourgogne-Franche Comté, Dijon, France; University of Shiga Prefecture, JAPAN

## Abstract

Carotenoids are diverse lipophilic natural pigments which are stored in variable amounts by animals. Given the multiple biological functions of carotenoids, such variation may have strong implications in evolutionary biology. Crustaceans such as *Gammarus* amphipods store large amounts of these pigments and inter-population variation occurs. While differences in parasite selective pressure have been proposed to explain this variation, the contribution of other factors such as genetic differences in the gammarid ability to assimilate and/or store pigments, and the environmental availability of carotenoids cannot be dismissed. This study investigates the relative contributions of the gammarid genotype and of the environmental availability of carotenoids in the natural variability in carotenoid storage. It further explores the link of this natural variability in carotenoid storage with major crustacean immune parameters. We addressed these aspects using the cryptic diversity in the amphipod crustacean *Gammarus fossarum* and a diet supplementation protocol in the laboratory. Our results suggest that natural variation in *G*. *fossarum* storage of dietary carotenoids results from both the availability of the pigments in the environment and the genetically-based ability of the gammarids to assimilate and/or store them, which is associated to levels of stimulation of cellular immune defences. While our results may support the hypothesis that carotenoids storage in this crustacean may evolve in response to parasitic pressure, a better understanding of the specific roles of this large pigment storage in the crustacean physiology is needed.

## Introduction

Carotenoids are diverse, lipophilic, biologically active natural pigments produced by photosynthetic micro-organisms, algae and plants. With rare exceptions, animals acquire carotenoids exclusively from their food [1–4]. Despite their great diversity in nature, few carotenoids are stored in animal tissues and fluids [5–7], suggesting that they are either selectively accumulated or metabolically transformed for their storage [8]. The amounts of carotenoids stored by animals vary across taxa [9,10] and within species [11], suggesting variability in their physiological importance, and/or fluctuations in their environmental availability, but the contributions of and interactions between genetics and environment remain not well understood.

Because they are involved in many biological functions [1,5], carotenoids may have strong implications in evolutionary biology, by impacting individual health, performance, and ultimately fitness. Their storage was suggested as indicative of individual quality, which could be used as sexually-selected traits [12], and they have beneficial effects on survival, growth, and immunity [5]. Besides being general immunostimulants, carotenoids also have the potential to scavenge free radicals produced by immune activity [13,14] and to interact with endogenous antioxidant enzymes [15–17]. While there is a strong controversy on the role antioxidant and immune stimulant activity of carotenoids in the avian literature [18–20], these effects appear more obvious in other systems such as marine and freshwater animals [21–25]. However, carotenoids were also suggested to have context-dependent detrimental effects when provided in excess under non-stressful conditions [26–28]. Beneficial effects of carotenoids were often secondarily derived from their conversion into downstream products, such as conversion of beta-carotene into vitamin A (crucial in the embryonic development), but also directly from non-provitamin A xanthophyll carotenoids such as astaxanthin and lutein, mainly produced by fungi, algae, and plants [2,29–31].

Xanthophyll carotenoids are stored in large amounts by aquatic animals, especially astaxanthin [8]. Crustaceans accumulate them as circulating lipid droplets in the haemolymph and as esterified forms in their tissues [7,32]. The precise reasons for such pigment accumulations in crustaceans remain unclear. This has not been linked to sexual selection so far but carotenoids have immunostimulating and antioxidant roles that may limit the immunopathology cost associated with the immune response [11,17,33]. However, large natural variation in the stored amounts of carotenoids was found among populations of a *Gammarus* crustacean amphipod [11]. The selective pressures or constraints leading to such a variation remain to be explained. Selection in response to differing parasitic pressures has been proposed to explain the inter-population variation in immune potential in these amphipods (levels of prophenoloxidase activity), which may, in turn, explain the inter-population variation in carotenoid accumulation [11,34]. Indeed, high prevalence of parasite attacks is accompanied by activation of the immune system. Individuals that improve their ability to store carotenoids, could then both enhance their immune responsiveness and reduce its associated immunopathology cost. Subsequently, these individuals may have a selective advantage compared to those which are not improving their carotenoid storage ability. However, confounding factors or local constraints such as inter-population variation in pigment availability in the crustacean environment (differences in food sources), or inter-population genetic differences in carotenoid assimilation and/or storage ability by the crustaceans cannot be dismissed.

The freshwater amphipod crustaceans of the genus *Gammarus* exhibit strong genetic differentiation among populations at regional and local scales [35]. In addition, the extensive use of DNA barcoding in recent years has allowed the detection of high degrees of cryptic diversity in gammarids, especially in *Gammarus pulex* and *G*. *fossarum* (that are genetically diverging lineages but which do not differ morphologically) [36–39]. The distribution of these cryptic lineages is not yet understood, since they are scattered among rivers at a large geographic scale, and often co-occur in sympatry in the same rivers or streams. Cryptic lineages of *G*. *fossarum* or *G*. *pulex* have been reported to show marked physiological, behavioral and ecological differences, sometimes at the level of interspecific differences [40,41]. For example, the different lineages are not equally susceptible to parasitism [42]. These lineages therefore provide an excellent and elegant biological model to test if the genetic background can be responsible for the natural variability in carotenoid storage in the haemolymph of crustaceans, and its link with major parameters of the cellular and humoral components of crustacean immunity (density of haemocytes, and activity of the prophenoloxidase cascade, an enzymatic cascade responsible for the melanisation response of invertebrate immunity). In this study, we sampled four natural populations of *G*. *fossarum*, and subjected them to a controlled diet supplementated with carotenoids. Chosen populations belong to two different cryptic genetic lineages showing 16% genetic divergence (among the 28 *G*. *fossarum* populations previously characterised by [38], by sequencing the mitochondrial cytochrome c oxidase subunit I) to test for the genotype component of carotenoid storage variability. For each lineage, replicate populations from two independent rivers were selected to test for the environmental component of this variability.

## Material and methods

### Experimental design and gammarid sampling

*Gammarus fossarum* (Crustacea: Amphipoda) were collected with a hand net in January 2014 in four independent rivers, thanks to the annual permit obtained for the Préfecture de Côte d’Or (last version: Arrêté Préfectoral n°77, 11/02/2019). The populations were chosen based on the results of the molecular study by [38], so that we sampled a set of two phylogenetically distant molecular operational taxonomic units (MOTUs) based on Cytochrome Oxidase subunit I mitochondrial marker (genetic divergence of ~16%, Kimura 2 parameters method) and two replicate populations within each MOTU (genetic divergence < 2%). We selected four populations of distinct and distant rivers where only a single MOTU has been detected: the populations of the rivers Doulonne (47°7'13.48” N, 5°44'14.60” E) and Norges (47°21'41.38” N, 5°9'30.16” E) for the MOTU *G*. *fossarum* I (referred to as Gf I, [38]), and the populations of the river Ource (47°46'59.01” N, 4°51'1.31” E) and Résurgence du Vivier (47°37'54.35” N, 5°30'36.79” E) for the MOTU *G*. *fossarum* VII (referred to as Gf VII) (S1 Fig). Within each MOTU, the two sampled populations were contrasted for abiotic and biotic ecological conditions. Several parameters of the sampled rivers, including the water level, presence of aquatic vegetation and dead leaves, the type of substrate on the river bottom, the presence of parasites, and the approximate gammarid density at the sampling points were assessed semi-quantitatively. This allowed us to assess if carotenoid storage was strictly linked to genetic proximity across different environmental conditions.

Throughout this study, gammarids were screened visually for the presence of acanthocephalan and microsporidian parasites. After field collection, parasitized gammarids were discarded from the following experiment but used to estimate the *in situ* prevalence of these common parasites. Gammarids that developed signs of an acanthocephalan or microsporidian infection during the experiment were excluded from the analyses because parasitism is known to depress immune parameters in infected individuals and high parasite prevalence would modulate immune parameters at the population level [34,43]. Therefore, the systematic discarding of parasitized gammarids avoids the potential confounding effect of parasitism on our measurements. For logistic reasons and to avoid sex effects (e.g. [44]), only males were used in this study. Gammarids were maintained individually in glass vials with 60 mL of aged water under standard laboratory conditions (15°C ± 1°C, light-dark cycle 12:12). To avoid a confounding effect of “local” ecological conditions, aged water was obtained by supplying with continuous oxygen for one week, a mix of dechlorinated and UV-sterilized tap water, with water and bottom substrate from a river where no gammarid was collected.

Immediately after field collection, the haemolymph of 30 individuals per population was sampled for further analyses (parameters at field collection; see below). The remaining gammarids were starved for 3 days to motivate feeding, and were then allocated to the two food treatments of the diet supplementation with carotenoids: half the gammarids received a diet without carotenoids, while the other half received a carotenoid-enriched diet (see details below). At two time points during the supplementation, 30 gammarids per treatment and population were sampled and their haemolymph was collected. The first sampling occurred 15 days of supplementation, which was previously reported to yield higher concentrations of circulating carotenoids in the haemolymph of supplemented gammarids [33]; see S1 Appendix. The second sampling occurred at 21 days of supplementation, to get close to the saturation in carotenoids of tissues and haemolymph; this is suggested by conspicuous changes in general body colouration observed already at 17 days of supplementation [17].

For each haemolymph sample (at field collection, and after 15 and 21 days of experimental treatments), the following parameters were measured: circulating carotenoids and two important crustacean immune parameters. We measured the density of haemocytes on half of the sampled gammarids, and the activities of the prophenoloxidase (proPO) cascade (natural PO activity, and total PO activity, see details below) on the other half of the samples. For each diet treatment and each sampled population, survival during diet supplementation was recorded on 40 additional gammarids. Gammarids were all weighted to the nearest mg, to control for individual fresh mass, using an OHAUS balance (discovery series, DU114C).

### Dietary supplementation with carotenoids

The food recipes were the same as in [33]. For both diet treatments, the food contained white fish meal (StarBAITS, Sensas, Fontenay-sur-Eure, France), wheat flour (Moulin Mecker-Diemer, Krautwiller, France), soy flour (l'Aliment Sain, Dijon, France), and vitamins (Premix Vitatech-S, AquaTechna, Couëron, France). The mixture was then moistened with either 780 mL of tap water (control diet treatment), or 780 mL of Oroglo solution containing 11g/kg of lutein and zeaxanthin (20:1, w/w, Kemin France, Nantes) to which 32.76 g of astaxanthin (Carophyll Pink 10%, DSM, Courbevoie) was added (supplemented diet treatment). This allowed reaching the ratio between astaxanthin and lutein of 4:1 observed in *Gammarus pulex* [7]. Sticks of about 5 mm diameter were modelled out of the dough, dried at 50°C for 4h, and stored at -80°C in aluminium foil. Gammarids were fed *ad libitum*. Remains of food provisions were removed when refreshing water of glass containers once a week.

### Haemolymph extraction, concentration of circulating carotenoids, and immune parameters

Haemolymph was collected with an ice-cooled glass capillary as described in [11]. The haemolymph sample of up to 3 μL was diluted in 20 μL of phosphate buffer saline (PBS: 8.74g NaCl, 1.78g Na_2_HPO_4_, 1000 mL of distilled water, pH 6.5), and split into a 10 μL sample for dosage of circulating carotenoids, and an up-to-13 μL sample for measurements of immune parameters (either the density of haemocytes, or the activities of the proPO cascade). Samples allocated to the carotenoids dosage and to the measurement of PO activities were snap-frozen and stored at -80°C. Samples allocated to the measurement of haemocyte density were screened immediately using a Neubauer-improved haemocytometer under a microscope (at magnification x 400).

Circulating carotenoids in the haemolymph were extracted and quantified using a microplate reader at 470 nm, as described in [11]. Sample concentration was determined against the reference curve of a standard solution of astaxanthin and lutein in ethanol (ratio 4:1; standards obtained from Extrasynthèse, Genay, France) with concentrations ranging from 0 to 50 ng/μL. Carotenoid concentrations were corrected to obtain concentrations for 1 μL of haemolymph. The activity of the naturally activated PO (hereafter, natural PO activity), and the activity of the naturally activated PO plus that of the pro-enzymes proPO hereafter, total PO activity) were measured with a spectrophotometric assay as described in [34]. Both activities were quantified on 5 μL of haemolymph extract in a microplate well by adding 20μL of PBS, and 140 μl of distilled water for natural PO activity or 140 μL of chymotrypsin (0.07 mg/mL solution; Sigma-Aldrich) for activation of pro-enzymes for total PO activity. For both activities, 20 μL of the substrate L-DOPA (4 mg/mL solution; Sigma-Aldrich), were added. The enzymatic reaction was recorded at 30°C for 40 min on a microplate reader (Versamax Molecular Devices). Enzyme activity (V_max_ in milliunit/min) was reported to the activity of 1 μL of pure haemolymph.

### Statistical analyses

Measures of field circulating carotenoids in the haemolymph, haemocyte density and activities of the proPO cascade were analysed with ANCOVA models, including the MOTU, the population nested within MOTU, the body mass as covariate, and their interactions. Data of parameters measured under controlled availability of carotenoids were analysed with the same models except that it included the diet treatment and the time point for sampling when necessary, and the interactions with these effects in addition to the other effects and the covariate. For the two sets of analyses, data were square-root transformed to meet the assumptions of residuals normality and homogeneity of variances of the analysis of variance. Non-significant effects or interactions were removed from the models. Data were analysed using JMP^®^ (SAS). Comparisons of the intensities of the response to the diet treatment, or comparisons of the parameters measured on gammarids from the field and those measured from gammarids after diet treatment were done with Cohen’s d (with 95% confidence intervals). Cohen’s d was also used on control and supplemented treatments to assess the effect size of diet treatment on measured parameters. Cohen’s d were calculated using the package effsize [45] in R software version 3.2.5 [46].

## Results

### Environmental conditions at the sampled sites

The four independent rivers where gammarids were sampled differed in several assessed biotic and abiotic conditions (Table 1). Especially, the four sites varied in their availability of aquatic vegetation and dead leaves, and on the presence and abundance of some crustacean parasites.

**Table 1:** General abiotic and biotic environmental characteristics at the four sampling sites recorded visually at the four sampling sites.

MOTU	Population	Water level	River bottom	Vegetation / dead leaves	Parasites
Gf I	Doulonne	< 30 cm	pebbles, stones, mud	+	+++
	Norges	> 30 cm	pebbles, stones	+	+
Gf VII	Ource	< 30 cm	sand	+++	+
	Vivier	> 30 cm	pebbles	+++	-

Several close small patches were sampled in each site, and the table provides an average value for each parameter. “-” corresponds to the absence of the considered parameter, “+” presence/low abundance, “++” presence/average abundance, “+++” presence/high abundance. Parasites presence is based on both visual observations on the sampling sites, and screening of sampled gammarids under a binocular dissecting microscope in the laboratory (see S1 Table. below).

None of the screened parasites was observed in gammarids of the population Résurgence du Vivier, while parasitized gammarids were found in the three other rivers (Table 2). Among these three latter rivers, gammarids of the population Doulonne and Ource showed the highest parasitism rates within their respective MOTU, essentially by microsporidia.

**Table 2:** Field parasite prevalence (%) in the sampled populations of the major parasites found in the four sampled populations and two MOTUs, the four acanthocephalan parasites *Pomphorhynchus laevis*, *P*. *tereticollis*, *Polymorphus minutus*, and *Echinorhynchus truttae*, and microsporidia, measured on sampled gammarids screened under a binocular dissecting microscope in the laboratory.

MOTU	Population	% parasitized	% Acanthocephala	% microsporidia	Screened males
Gf I	Doulonne	26 %	1 %	25 %	336
	Norges	7 %	6 %	1 %	268
Gf VII	Ource	9 %	0 %	9 %	269
	Vivier	0 %	-	-	265

### Variation in concentrations of circulating carotenoids between and within MOTUs

**Fig 1:**
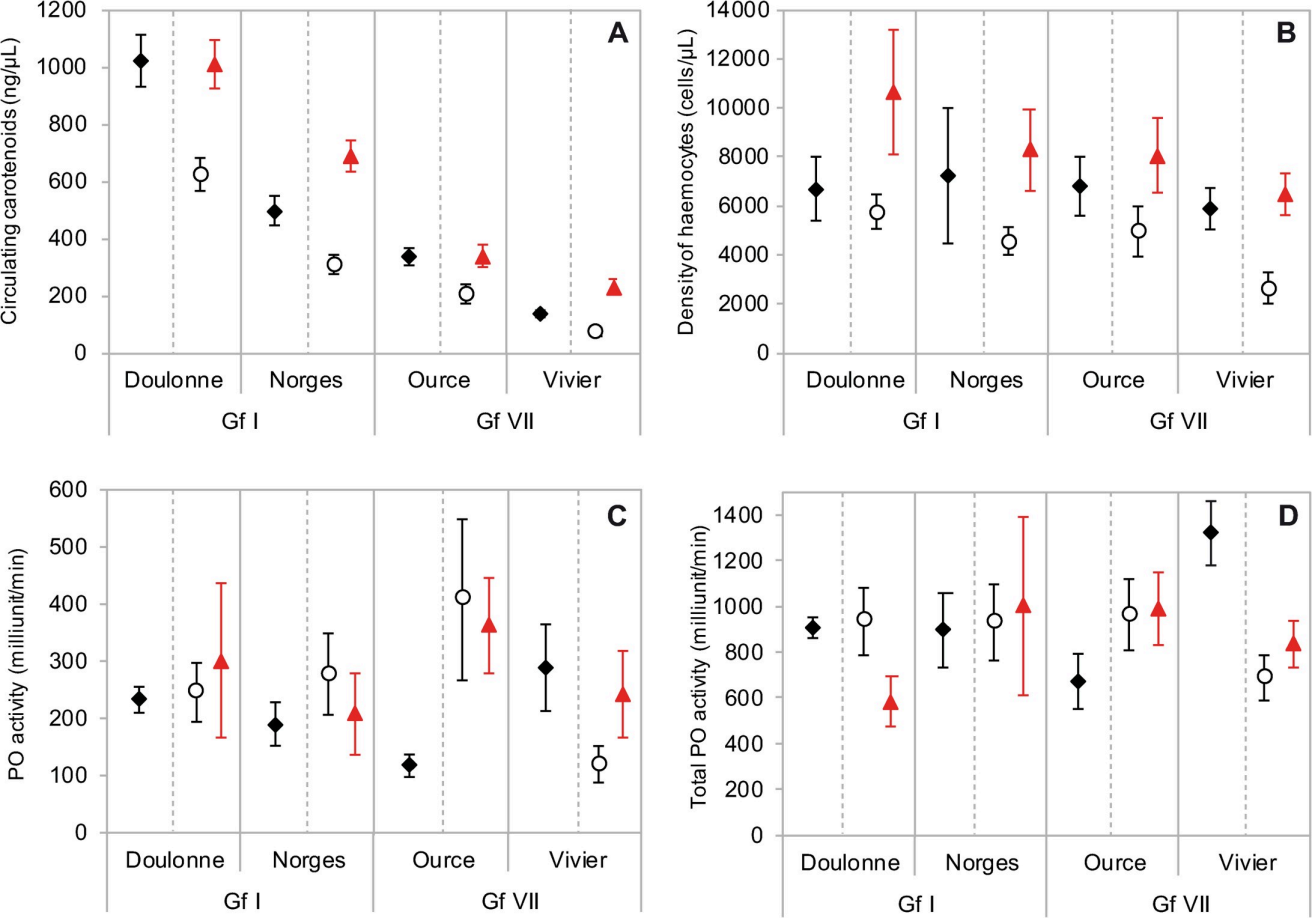
(A) Concentration of circulating carotenoids, (B) density of haemocytes, (C) natural PO activity and (D) total PO activity of the haemolymph of gammarids under field conditions (black filled lozenges, left symbols), and after 21 days of diet supplementation with carotenoids for non-supplemented control gammarids (open circles) and supplemented gammarids (red triangles), for the two populations within each of the two MOTUs of *Gammarus fossarum* (Gf I and Gf VII). All values are mean ± se. For each population, sampling, and diet treatment, *N* = 30 gammarids for carotenoid concentrations, *N* = 15 gammarids for each immune parameter.

In the field, concentrations of circulating carotenoids in the haemolymph varied both between and within MOTUs. Gf I gammarids stored greater amounts of carotenoids in their haemolymph than Gf VII gammarids (Fig 1A, Table 3). Within each MOTU, carotenoid concentrations differed between gammarid populations (Table 3). Gammarids collected in the populations Doulonne and Ource showed the highest carotenoid contents for MOTU Gf I and Gf VII, respectively. Gammarid body mass had no effect on carotenoid storage in the haemolymph (Table 3), although individuals of the population Résurgence du Vivier weighted more than twice as much as those of the three other populations but had the lowest pigment storage (S1 Table).

**Table 3:** Results of the ANCOVA analysis on the concentrations of circulating carotenoids, density of haemocytes, and natural and total PO activities in the haemolymph, in the field and at 21 days of diet supplementation in the laboratory.

Source of variation	*df*	*F* ratio	*P* value
**In the field :**			
*- Concentrations of circulating carotenoids*			
Model	4,111	46.90	**<0.0001**
MOTU	1,111	49.58	**<0.0001**
Population[MOTU]	2,111	26.35	**<0.0001**
Body mass	1,111	0.06	0.80
Body mass × MOTU	1,108	3.40	0.07
Body mass × Population[MOTU]	2,108	2.04	0.13
*- Density of haemocytes*			
Model	5,52	1.88	0.11
MOTU	1,52	7.22	0.01
Population[MOTU]	2,52	1.48	0.24
Body mass	1,52	8.81	0.005
*- Natural PO activity*			
Model	4,52	1.97	0.11
MOTU	1,52	0.75	0.39
Population[MOTU]	2,52	3.57	0.04
Body mass	1,52	2.19	0.15
*- Total PO activity*			
Model	4,52	2.71	**0.04**
MOTU	1,52	0.38	0.54
Population[MOTU]	2,52	2.75	0.07
Body mass	1,52	0.08	0.78
**After 21 days of diet supplementation with carotenoids :**			
*- Concentrations of circulating carotenoids*			
Model	8,229	43.63	**<0.0001**
MOTU	1,229	94.6	**<0.0001**
Population[MOTU]	2,229	10.86	**<0.0001**
Diet treatment	1,229	72.20	**<0.0001**
Body mass	1,229	18.48	**<0.0001**
Diet treatment × MOTU	1,225	0.25	0.62
Diet treatment × Population[MOTU]	2,225	2.35	0.10
Diet treatment × Body mass	1,229	0.14	0.71
Body mass × MOTU	1,229	6.02	**0.015**
Body mass × Population[MOTU]	2,229	8.40	**0.0003**
*- Density of haemocytes*			
Model	5,114	6.79	**<0.0001**
MOTU	1,114	0.77	0.38
Population[MOTU]	2,114	1.07	0.35
Diet treatment	1,114	20.49	**<0.0001**
Body mass	1,114	1.64	0.20
Diet treatment × MOTU	1,107	2.14	0.15
Diet treatment × Population[MOTU]	2,107	1.45	0.24
Diet treatment × Body mass	1,107	2.61	0.11
Body mass × MOTU	1,107	1.54	0.22
Body mass × Population[MOTU]	2,107	0.30	0.74
*- Natural PO activity*			
Model	5,114	2.29	0.051
MOTU	1,114	3.58	0.06
Population[MOTU]	2,114	0.16	0.86
Diet treatment	1,114	0.06	0.81
Body mass	1,114	5.57	0.02
*- Total PO activity*			
Model	5,112	0.60	0.70
MOTU	1,114	0.56	0.46
Population[MOTU]	2,114	0.45	0.64
Diet treatment	1,114	0.59	0.44
Body mass	1,114	0.09	0.76

Significant *P* values are highlighted in bold.

**Fig 2:**
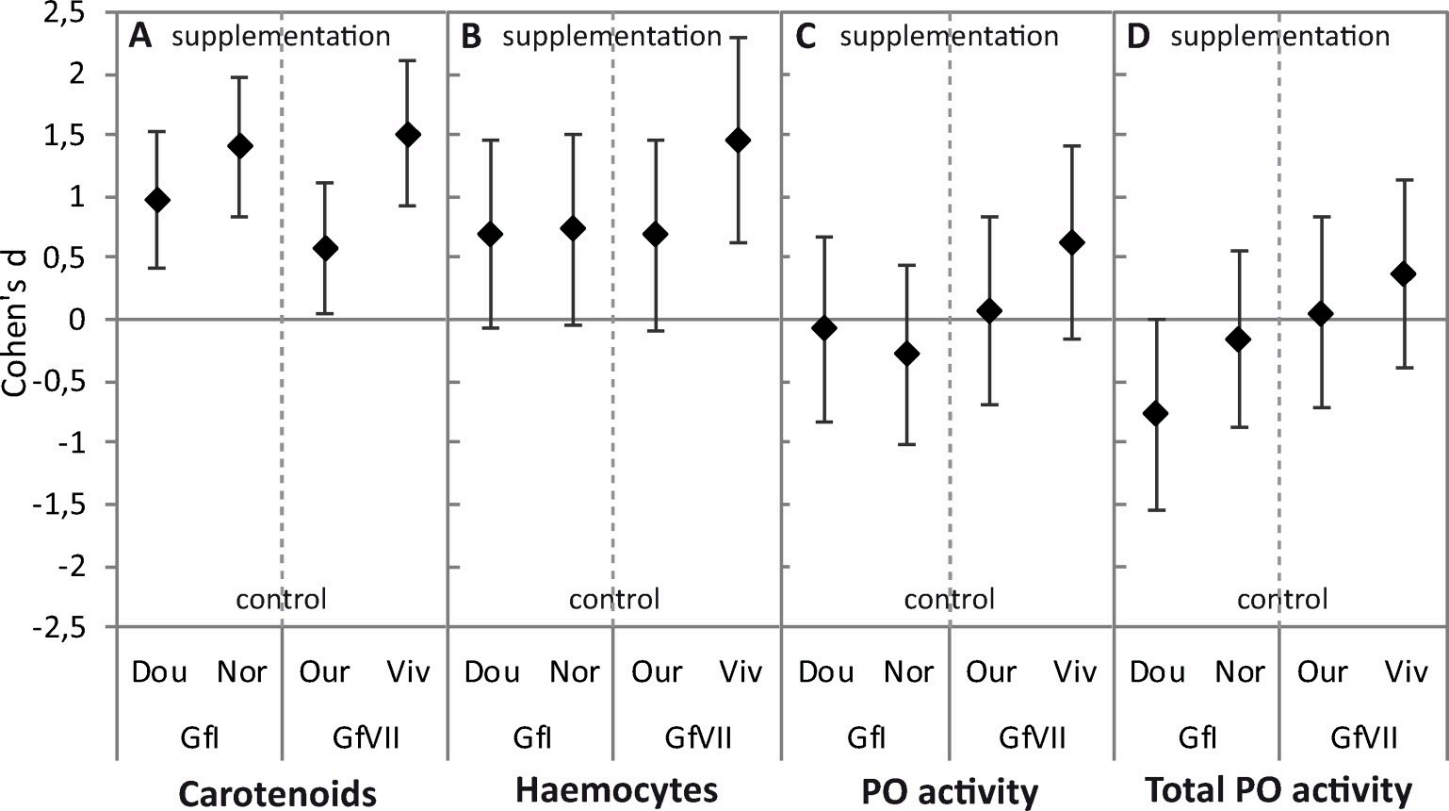
Cohen’s d and 95% confidence intervals for diet treatment effect (supplementation *vs*. control) on (A) the concentration of circulating carotenoids, (B) the density of haemocytes, and (C, D) the natural and total PO activities respectively, after 21 days of diet supplementation with carotenoids in the laboratory. Significant effect size appears when the 95% CI does not include 0. For each population and diet treatment, *N* = 30 gammarids for carotenoid concentrations, *N* = 15 gammarids for each immune parameter. Dou: Doulonne, Nor: Norges, Our: Ource, and Viv: Résurgence du Vivier.

**Fig 3:**
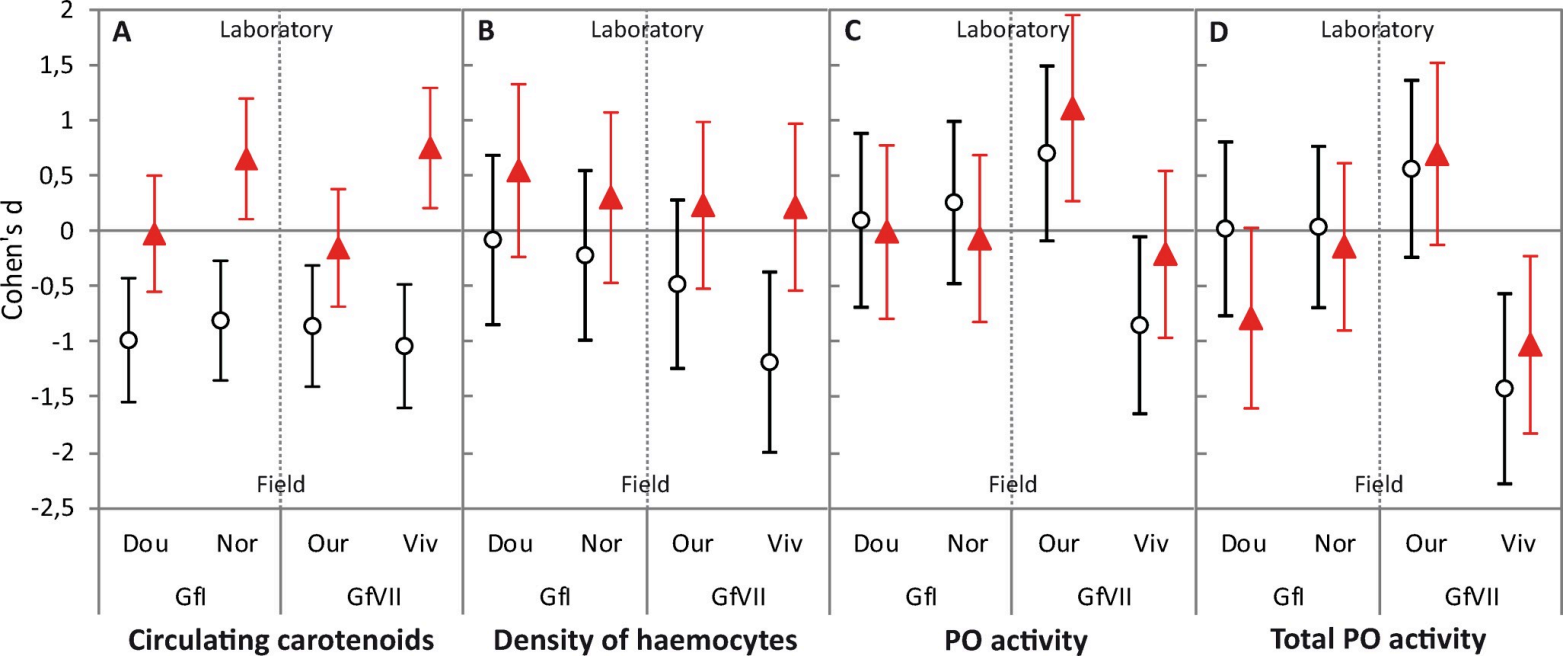
Cohen’s d and 95% confidence intervals for the comparisons of values of laboratory conditions *vs*. field conditions for (A) the concentration of circulating carotenoids, (B) the density of haemocytes, and (C, D) the natural and total PO activities respectively. Gammarids supplemented with carotenoids (21 days) in the laboratory are red triangles and non-supplemented control gammarids are open circles. Significant effect size appears when the 95% CI does not include 0. For each population, sampling, and diet treatment, *N* = 30 gammarids for carotenoid concentrations, *N* = 15 gammarids for each immune parameter. Dou: Doulonne, Nor: Norges, Our: Ource, and Viv: Résurgence du Vivier.

After 21 days of diet supplementation with carotenoids in the laboratory, carotenoid concentrations in the haemolymph still varied both between and within MOTUs, the ranks of MOTUs and populations observed in the field being conserved (Fig 1A, Table 3). For the two MOTUs and the four sampled populations, the diet treatment yielded higher carotenoid concentrations in the haemolymph of supplemented gammarids than in that of non-supplemented controls (Fig 1A, Table 3). The size of the supplementation effect was quite similar between populations of the MOTU Gf I, but it was higher in the population of Résurgence du Vivier than in the population of Ource within the MOTU Gf VII (Fig 2A). Interestingly, values recorded for supplemented gammarids fell within the natural range of concentrations observed in the field for the two populations showing higher carotenoid concentrations within their MOTU (Gf I Doulonne, and Gf VII Ource; Cohen’s d close to 0), while values of supplemented gammarids of the two lower populations exceeded those observed in the field (Gf I Norges, and Gf VII Résurgence du Vivier; Cohen’s d significantly above 0) (Figs 1A and 3). By contrast, all the non-supplemented groups of gammarids showed lower carotenoid concentrations than those observed in the field, with similar effect size for the four populations (Fig 3). Similar patterns of carotenoids storage in supplemented and control gammarids were obtained at 15 days of diet supplementation, with smaller effect sizes (S1 Appendix).

Upon diet supplementation, gammarid body mass positively influenced the concentrations of circulating carotenoids: greater amounts of carotenoids were stored by heavier gammarids of the two populations of MOTU Gf I and the population Ource of MOTU Gf VII. However, fewer carotenoids were stored by heavier gammarids of the population Résurgence du Vivier of MOTU Gf VII, which are by far the heaviest among the collected crustaceans (Table 3 and S1 Table). This body mass effect occurred regardless of the diet treatment (Table 3). Similar body mass effects were obtained at 15 days of diet supplementation with carotenoids (S1 Appendix). No difference in mortality between supplemented and control gammarids was detected during diet supplementation (S2 Table).

### Variation in immune parameters between and within MOTUs

In the field, the density of haemocytes did not vary significantly between and within gammarid MOTUs (Fig 1B, Table 3; the model does not fit significantly the data), nor did the natural PO activity and the total PO activity (Fig 1C and 1D, Table 3). Although not statistically significant, the heavy gammarids of the population Résurgence du Vivier (S1 Table) showed higher values for both PO activities.

After 21 days of diet supplementation in the laboratory, in line with the results of carotenoids concentrations, the diet treatment yielded higher density of haemocytes in supplemented gammarids than in non-supplemented controls for all the populations and MOTUs. The effect size of supplementation was nevertheless larger for gammarids of population Résurgence du Vivier (Figs 1B and 2B, Table 3). Values for supplemented gammarids fell within the natural range of values observed in the field, as were the haemocyte counts for most non-supplemented animals (Fig 3). However, lower densities were found in non-supplemented gammarids of Résurgence du Vivier compared to animals caught in the field (Fig 3). The density of haemocytes was still not significantly variable between and within MOTUs (Fig 1B, Table 3). Finally, the gammarid body mass had no effect on this immune parameter (Table 3).

By contrast, variation in both natural PO activity and total PO activity was not explained by either the diet treatment, nor the gammarid MOTU and population, nor the body mass (Fig 1C and 1D and Fig 2, Table 3; the models do not fit significantly the data for both PO activities). Nevertheless, PO activities measured after 21 days of diet treatment remained within the natural range of values observed in the field, except for a higher effect size of supplementation on natural PO activity in gammarids from Ource and a lower effect size on PO activities in gammarids from population Résurgence du Vivier (Fig 3). At 15 days of diet supplementation, results were very similar, with generally no main effect of the diet treatment, the gammarid MOTU or population, or the body mass, in the observed variation (S1 Appendix).

## Discussion

### Variation in carotenoid levels

In the field, variation in the storage of dietary carotenoids in the gammarid haemolymph occurred naturally, as previously reported in the correlative study by [11]. Whether such a variation could be adaptive to support oxidative costs of immune defences against frequent parasite attacks [11], and results from local availability of the pigments in the field and/or relies on large cryptic genetic differences among populations is here discussed. Gammarids of the two *Gammarus fossarum* lineages (MOTUs) Gf I and Gf VII stored different amounts of carotenoids in their haemolymph, Gf I gammarids storing greater amounts of pigments. These two lineages exhibit high genetic divergence (~16% based on the mitochondrial barcoding marker COI analysis) and seldom form hetero-genotypes pairs when coexisting in sympatry in the field, and hardly breed in the laboratory [38]. Despite their similar morphology, this allows considering them as two *G*. *fossarum* cryptic species, or at least genotypes in the course of speciation [38]. This kind of cryptic variation (in what was previously thought to be a single species) may therefore explain a part of the variation in the gammarids’ ability to store carotenoids. Gammarids are both shredders and predators [47,48], and the different gammarid cryptic species may also differ in their foraging and predatory behaviour, therefore differing in the sources of carotenoids they get access to from their diet. Cryptic speciation is a general phenomenon in gammarids [38,49,50], and certainly explains most of the physiological variation observed in this taxonomic group [40, 42]. Genetic divergence within cryptic species (around 2%; [38]) likely accounts for an additional part of this variation.

Field concentrations of circulating carotenoids varied between populations within each of these cryptic species. The population sites were sampled all at the same time, and are located in the same region of France, ruling out possible confounding seasonal and macro-scale fluctuations of carotenoid availability. Differences in ecological conditions between sampled sites should therefore influence carotenoid availability, and may account for an additional part of the observed variation in field carotenoid storage. This conclusion is strengthened by the observation that gammarids from the two populations with the lowest carotenoid contents, within each cryptic species, showed an increase in their carotenoid contents after supplementation, above their carotenoid contents in the field, while such an increase was not observed in populations exhibiting the highest levels of carotenoids. Some local conditions in the field may limit the gammarids’ ability to acquire and/or store pigments (in the former populations), but not in others (in the later populations). Developmental conditions such as the amounts of carotenoids in the developmental environment (i.e. in the egg), which depends directly on the pigments’ availability, could influence the post-hatching metabolism of carotenoids and hence the ability to acquire and store these pigments later in life, as was reported in birds [51]. In the frame of this study, this could have impacted the gammarid ability to acquire and/or store carotenoids during a controlled supplementation of the diet with carotenoids. From our rough estimates of ecological conditions, the magnitude of circulating carotenoid concentrations of the populations might not be strongly influenced by the abundance of decaying plant material in the stream on which gammarids feed as shredders [47]. Not quantified here, differences in microbial communities and algae, which also produce carotenoids [2] and are a diet source for gammarids [47], may contribute to the differences in field carotenoid storage between populations. Finally, the different local conditions may include variable amounts of prey available, which could contribute to the variation in the sources of carotenoids that gammarids can acquire from their diet.

It is noteworthy that within each cryptic species, the populations maintaining the highest concentrations of carotenoids in the field (Doulonne and Ource) are also those locally exposed to a relatively stronger parasite pressure, as revealed by the parasite prevalence observed (Doulonne 26% *vs*. Norges 7%, and Ource 9% *vs*. Résurgence du Vivier 0%; Table 2). Furthermore, gammarids from these populations appear to store a maximum of carotenoids in their haemolymph either in the field or in the laboratory upon supplementation, whereas the others were able to store more carotenoids upon supplementation than in the field. Frequent challenges by parasites induce oxidative stress through enhanced immune activity, and carotenoids are concomitantly consumed by the immune response, probably to scavenge the resulting excess of free radicals [17]. Such a stress could even be stronger when gammarids are suffering from additional environmental stressors, which are often prevalent in nature. For instance, parasitized *Gammarus roeseli* exhibited a strong depletion of circulating carotenoids and enhanced level of lipoperoxidation damages when exposed to pollutants [52,53]. Similar states of stress might be possible from environmental temperature changes when combined with pathogenic challenges [54]. Our results therefore appear consistent, at both the within and between cryptic species levels, with the hypothesis that evolving greater capacity of carotenoid storage in response to the necessity of increasing immune activity to deal with higher parasitic threat could be adaptive [11]. Here, gammarids from the populations of Doulonne and Ource exhibited the highest capacity of carotenoids storage and tended to keep storage levels at maximum in the field. This might be advantageous when dealing with prevalent parasite infections. Data from additional gammarid populations exposed to contrasted prevalence of pathogen/parasite attacks are required to be conclusive.

Consistent with previous studies on *G*. *pulex* [17,33], carotenoid concentrations increased upon supplementation in the haemolymph of supplemented gammarids for all the cryptic species and populations compared to their respective non-supplemented controls (in addition, the longer the supplementation, the larger the increase in carotenoid storage, see Electronic Supplementary Material). Compared to concentrations in the field, carotenoid concentrations during supplementation changed differently depending on the diet treatment and the gammarid population within cryptic species. All non-supplemented gammarids showed a depletion of circulating carotenoids in their haemolymph. Maintenance under laboratory conditions is stressful for crustaceans [55], and stress can deplete carotenoid stocks [56]. Non-supplemented gammarids may have therefore consumed the carotenoids they had previously stored from their natural habitat, to cope with the stress induced by laboratory maintenance. The applied stress level being identical for all the gammarids, this may partially account for the persistent variation between populations. In contrast, carotenoid concentrations of supplemented gammarids were not lowered during laboratory maintenance. This shows that the supplementation prevented the carotenoids depletion induced by laboratory conditions, and likely help gammarids to better cope with stress, as reported in crustaceans of commercial interest [22,23] and *G*. *pulex* [33]. Nevertheless, here this did not translate into a survival benefit (Electronic Supplementary Material).

During supplementation, circulating carotenoid concentrations co-varied positively with gammarid body mass, indicating that heavier individuals had a greater assimilation and/or storage capacity than lighter ones. Surprisingly, this did not remain consistent for gammarids of the two Gf VII populations in which heavier individuals stored fewer pigments, and especially for the population Résurgence du Vivier in which gammarids were by far the heaviest. This could be due to within-species genetic divergence and to the cryptic species genetic background.

### Variation in immune parameters

In the field, the density of haemocytes and the levels of activity (natural PO activity) and maintenance (total PO activity) of the prophenoloxidase (ProPO) cascade did not vary between or within cryptic species. This is surprising given the (rough) estimates of prevalence of common gammarid parasites, especially in the case of the population Résurgence du Vivier, in which no parasite was found and for which the lowest levels of immune parameters may have been expected. Indeed, the selection pressure applied by parasites may generate between-population variation in immune parameters ([34], see above). This is not the case here, and this is especially visible for the population Résurgence du Vivier. This lack of concordance may be due to the specificity of this latter population, for which many of the investigated parameters are quite intriguing (large size of the gammarids, high natural PO activity and maintenance, decreasing of total PO activity after carotenoid supplementation (see below)), and remain to be explained.

The supplementation with carotenoids yielded higher density of haemocytes in supplemented gammarids, mainly at the end of the diet treatment, and within the range of values in the field. This is consistent with previous reports of stimulation of constitutive levels of crustacean immune defences by diet supplementation with carotenoids, especially with astaxanthin [23,57]. However, the supplementation had no effect on the activity or the maintenance of the proPO cascade. In gammarids, previous studies reported that the resistance to bacterial infection is broadly stimulated by diet supplementation with carotenoids, but the particular stimulated immune effectors are not systematically the same depending on the sampled populations, being once the density of haemocytes and once the activity of the proPO cascade [17,33]. Interestingly, in our study, the effect of the supplementation on density of haemocytes was clearly larger for the population Résurgence du Vivier, which goes with its larger response to the supplementation for carotenoid concentrations (although it still had the lowest concentrations among sampled populations). However, this larger supplementation effect results mainly from the stronger decrease in this immune parameter in non-supplemented gammarids, outside the range of field values.

To summarize, our field collections and the diet supplementation with carotenoids in the laboratory showed that the natural variability in the storage of dietary carotenoids in the haemolymph is explained by the interplay between the environmental availability of carotenoids and the genetically-determined gammarid ability to assimilate and, especially, to store these pigments. *Ad libitum* availability of carotenoids under controlled laboratory conditions confirmed the stimulating power on gammarid cellular immune defences. It also revealed the importance of genotype in the persistent natural variation in carotenoid storage in *G*. *fossarum*. Further investigations, including additional cryptic species and populations within species, as well as other gammarid species (such as the invasive *G*. *roeseli*), and the quantitative assessment of environmental availability of carotenoids in the field would provide additional data to explore more deeply the origin of natural variability in the storage of large amounts of dietary carotenoids in crustaceans. In particular, investigations among populations that are locally exposed to contrasted parasite pressure should provide important insights to test the hypothesis that the evolution of carotenoid storage is driven by parasitism. To this purpose, the *G*. *fossarum*-*G*. *pulex* species complex is an ideal system to perform a comparative phylogenetic study that would help draw more solid conclusions on the origin of this natural variability. It would further help better understand the special roles of this large pigment storage in the crustacean physiology.

## Supporting information

S1 Fig Map of the sampling sites. (DOCX)

S1 Appendix Carotenoid concentrations and immune parameters measured on the hemolymph of gammarids at 15 days of diet supplementation with carotenoids. (DOCX)

S1 Table Body mass of sampled gammarids. (DOCX)

S2 Table Mortality of gammarids during diet supplementation. (DOCX)
