# Supplementary material for: Origin of the natural variation in the storage of dietary carotenoids in freshwater amphipod crustaceans
Source: PLoS One. 2020 Apr 15;15(4):e0231247. doi: 10.1371/journal.pone.0231247 (PMC7159244; doi:10.1371/journal.pone.0231247)
Supplement: S1 Fig — (DOCX) [file pone.0231247.s001.docx]

**S1 Figure. Map of the sampling sites.** Location of the four sampling sites of gammarids in the North-East part of France. The populations of the rivers Doulonne and Norges belong to the MOTU *G. fossarum* I, and the populations of the river Ource and of the Résurgence du Vivier belong to the MOTU *G. fossarum* VII.
