# Supplementary material for: Origin of the natural variation in the storage of dietary carotenoids in freshwater amphipod crustaceans
Source: PLoS One. 2020 Apr 15;15(4):e0231247. doi: 10.1371/journal.pone.0231247 (PMC7159244; doi:10.1371/journal.pone.0231247)
Supplement: S1 Appendix — (DOCX) [file pone.0231247.s002.docx]

**S2 Appendix. Carotenoid concentrations and immune parameters measured on the haemolymph of gammarids at 15 days of diet supplementation with carotenoids**

At 15 days of diet supplementation, gammarid haemolymph was collected as described in the main article, to quantify circulating carotenoids and measure the density of haemocytes and the natural and total PO activities.

*Variation in concentrations of circulating carotenoids between and within MOTUs*

Carotenoid concentrations in the haemolymph were generally lower than those obtained at 21 days of diet treatment, especially for supplemented gammarids (Figure S2.1A, Table S2.1). As later during the diet treatment, the supplementation with carotenoids yielded already higher carotenoid concentrations in supplemented gammarids than in non-supplemented controls (Table S2.2). The effect sizes were smaller than at 21 days of supplementation and significant only for the two populations showing the lowest concentrations within their MOTU (Norges for Gf I, and Résurgence du Vivier for Gf VII; Figure S2.2). Variation between and within MOTUs occurred as at 21 days of diet treatment, with greater carotenoid concentrations in Gf I gammarids, and the same population ranks within each MOTU (Figure S2.1A; Table S2.2). Compared to field values, non-supplemented controls of the four populations and the two MOTUs had generally lower carotenoid concentrations, while supplemented gammarids had similar concentrations (Figure S2.3).

Gammarid body mass influenced the circulating carotenoids in the haemolymph, but depending on the sampled MOTU: heavier individuals of MOTU Gf I and lighter individuals of MOTU Gf VII had higher carotenoid concentrations (Table S2.2). By contrast with the sampling at 21 days of diet treatment, the populations within MOTU Gf VII did not differ yet in their body mass effect (Table S2.2).

*Variation in immune parameters between and within MOTUs*

At 15 days of diet supplementation, as for carotenoid concentrations, the density of haemocytes was generally lower than at 21 days of supplementation, but essentially for supplemented gammarids (Figure S2.1B, Table S2.1). The diet treatment had no effect yet on this immune parameter (Figure S2.2, Table S2.2). However, while the observed variation was not explained by the MOTU (Table S2.2), populations within MOTUs differed almost as at 21 days of diet treatment (Table S2.2): greater densities of haemocytes were measured for gammarids of population Ource compared to Résurgence du Vivier within MOTU Gf VII, but no significant difference was detected between gammarid populations within MOTU Gf I (Figure S2.1B). Body mass had no effect on this immune parameter (Table S2.2).

For the activities of the proPO cascade, values at 15 days of diet treatment were generally lower than values at 21 days of treatment for natural PO activity only (Figure S2.1C and D, Table S2.1). Variation in natural and total PO activities was not explained by the MOTU, the population within MOTU, or the diet treatment (Figures S2.1C and D, Table S2.2). Only the gammarid body mass influenced the natural PO activity: heavier gammarids had lower enzyme activity (Table S2.2).

**Table S2.1.** Results of the ANCOVA analysis of the dynamics of concentrations of circulating carotenoids, density of haemocytes, and natural and total PO activities measured at 15 days and 21 days of diet supplementation in the laboratory.

| **Source of variation** | ***df*** | ***F* ratio** | ***P* value** |
| --- | --- | --- | --- |
| *- Concentrations of circulating carotenoids* |  |  |  |
| Model | 9,466 | 60.86 | **<0.0001** |
| Time | 1,466 | 6.94 | **0.009** |
| Time × MOTU | 1,457 | 1.00 | 0.32 |
| Time × Population[MOTU] | 2,457 | 2.07 | 0.13 |
| Time × Diet treatment | 1,457 | 1.78 | 0.18 |
| Time × Body mass | 1,457 | 0.96 | 0.33 |
| *- Density of haemocytes* |  |  |  |
| Model | 7,229 | 8.15 | **<0.0001** |
| Time | 1,229 | 5.09 | **0.025** |
| Time × MOTU | 1,218 | 0.03 | 0.87 |
| Time × Population[MOTU] | 2,218 | 1.44 | 0.24 |
| Time × Diet treatment | 1,229 | 9.74 | **0.002** |
| Time × Body mass | 1,218 | 0.27 | 0.60 |
| *- Natural PO activity* |  |  |  |
| Model | 6,232 | 6.70 | **<0.0001** |
| Time | 1,232 | 16.59 | **<0.0001** |
| Time × MOTU | 1,220 | 0.81 | 0.37 |
| Time × Population[MOTU] | 2,220 | 0.19 | 0.82 |
| Time × Diet treatment | 1,220 | 1.22 | 0.27 |
| Time × Body mass | 1,220 | 0.23 | 0.63 |
| *- Total PO activity* |  |  |  |
| Model | 6,228 | 1.50 | 0.18 |

**Table S2.2.** Results of the ANCOVA analysis on the concentrations of circulating carotenoids, density of haemocytes, and natural and total PO activities in the haemolymph at 15 days of diet supplementation in the laboratory.

| **Source of variation** | ***df*** | ***F* ratio** | ***P* value** |
| --- | --- | --- | --- |
| *- Concentrations of circulating carotenoids* |  |  |  |
| Model | 6,231 | 38.90 | **<0.0001** |
| MOTU | 1,231 | 75.10 | **<0.0001** |
| Population[MOTU] | 2,231 | 26.50 | **<0.0001** |
| Diet treatment | 1,231 | 27.40 | **<0.0001** |
| Body mass | 1,231 | 11.46 | **0.0008** |
| Diet treatment × MOTU | 1,225 | 0.18 | 0.67 |
| Diet treatment × Population[MOTU] | 2,225 | 0.54 | 0.59 |
| Diet treatment × Body mass | 1,225 | 0.08 | 0.78 |
| Body mass × MOTU | 1,231 | 7.21 | **0.008** |
| Body mass × Population[MOTU] | 2,225 | 0.43 | 0.65 |
| *- Density of haemocytes* |  |  |  |
| Model | 5,111 | 4.35 | **0.001** |
| MOTU | 1,111 | 0.52 | 0.47 |
| Population[MOTU] | 2,111 | 6.61 | **0.002** |
| Diet treatment | 1,111 | 0.05 | 0.83 |
| Body mass | 1,111 | 0.08 | 0.78 |
| Diet treatment × MOTU | 1,104 | 0.17 | 0.69 |
| Diet treatment × Population[MOTU] | 2,104 | 2.04 | 0.14 |
| Diet treatment × Body mass | 1,104 | 0.66 | 0.42 |
| Body mass × MOTU | 1,104 | 0.15 | 0.70 |
| Body mass × Population[MOTU] | 2,104 | 2.39 | 0.10 |
| *- Natural PO activity* |  |  |  |
| Model | 5,113 | 4.15 | **0.002** |
| MOTU | 1,113 | 1.57 | 0.21 |
| Population[MOTU] | 2,113 | 0.67 | 0.51 |
| Diet treatment | 1,113 | 2.88 | 0.09 |
| Body mass | 1,113 | 11.86 | **0.0008** |
| Diet treatment × MOTU | 1,106 | 1.08 | 0.30 |
| Diet treatment × Population[MOTU] | 2,106 | 1.63 | 0.20 |
| Diet treatment × Body mass | 1,106 | 1.09 | 0.30 |
| Body mass × MOTU | 1,106 | 0.64 | 0.42 |
| Body mass × Population[MOTU] | 2,106 | 0.58 | 0.56 |
| *- Total PO activity* |  |  |  |
| Model | 5,110 | 1.94 | 0.08 |


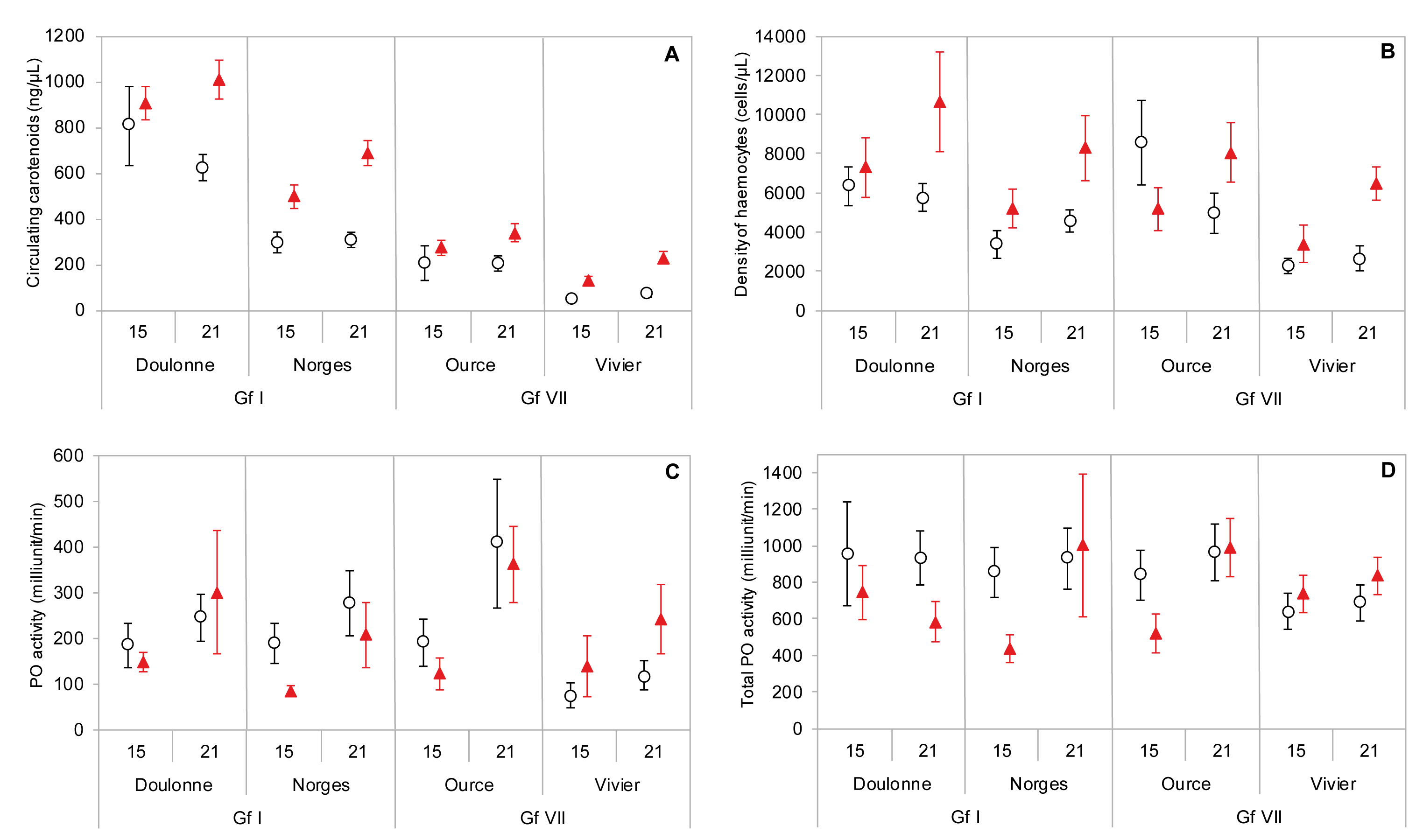


**Figure S2.1.** Concentration of circulating carotenoids (A), density of haemocytes (B), natural PO activity (C) and total PO activity (D) in the haemolymph of non-supplemented control gammarids (open circles) and supplemented gammarids (red triangles) at 15 days and 21 days of diet supplementation with carotenoids, for the four populations and the two MOTUs Gf I and Gf VII (mean ± se). For each population, sampling, and diet treatment, *N* = 30 gammarids for carotenoid concentrations, *N* = 15 gammarids for each immune parameter.


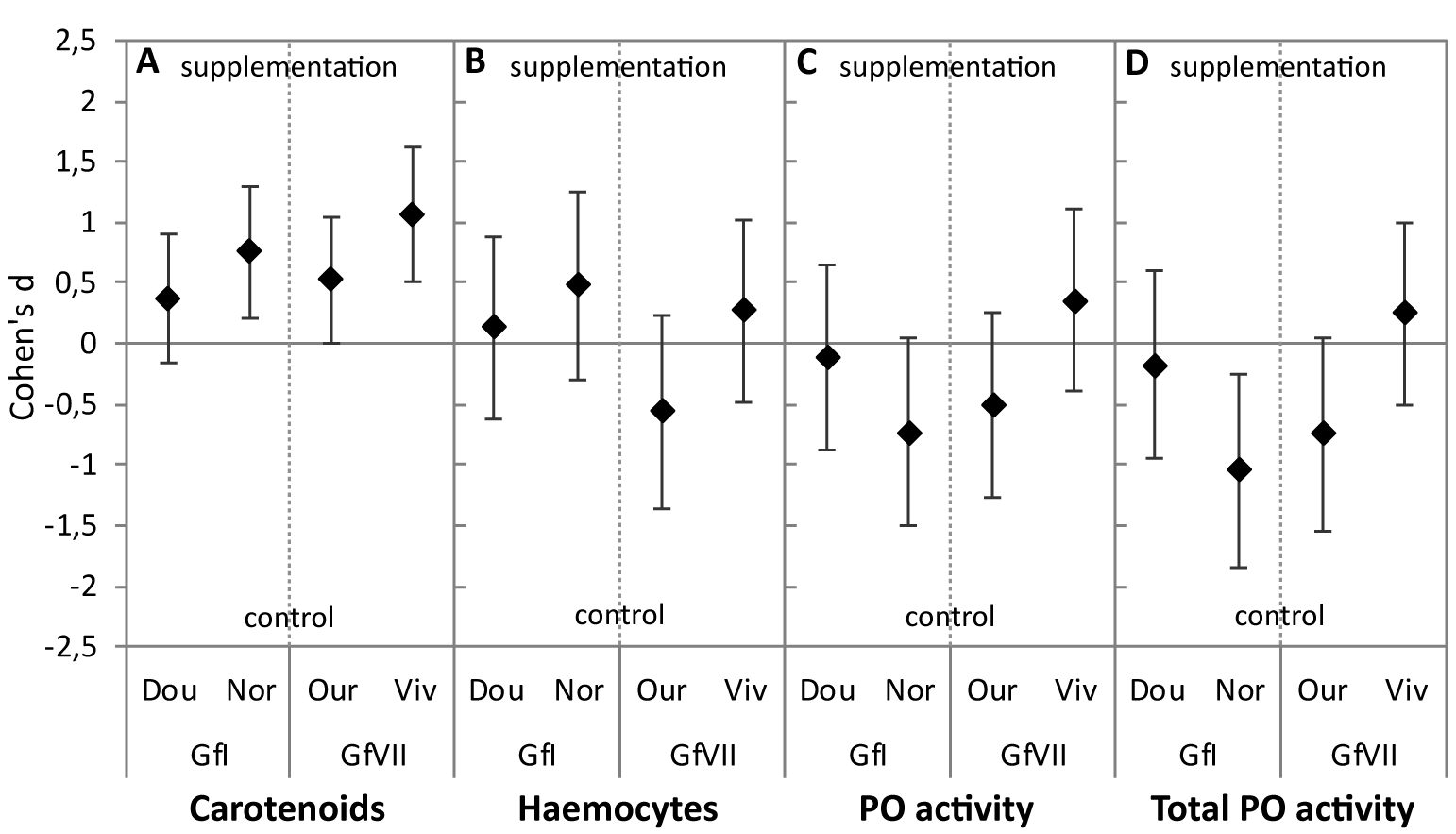


**Figure S2.2.** Cohen’s d and 95% confidence intervals for diet treatment effect (supplementation *vs.* control) on concentrations of circulating carotenoids (A), density of haemocytes (B), and natural and total PO activities (C, D respectively) after 15 days of diet supplementation with carotenoids. Significant effect size significantly appears when the 95% CI does not include 0. For each population and diet treatment, *N* = 30 gammarids for carotenoid concentrations, *N* = 15 gammarids for each immune parameter. Dou: Doulonne, Nor: Norges, Our: Ource, Viv: Résurgence du Vivier


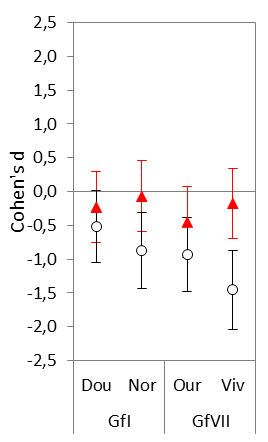


**Figure S2.3.** Cohen’s d and 95% confidence intervals for circulating carotenoids to compare values of supplemented gammarids (red triangles) and non-supplemented controls (open circles) after 15 days of diet carotenoid supplementation, with values in field conditions. Significant effect size appears when the 95% CI does not include 0. For each population, and diet treatment, *N* = 30 gammarids. Dou: Doulonne, Nor: Norges, Our: Ource, and Viv: Résurgence du Vivier.
