# Supplementary material for: Origin of the natural variation in the storage of dietary carotenoids in freshwater amphipod crustaceans
Source: PLoS One. 2020 Apr 15;15(4):e0231247. doi: 10.1371/journal.pone.0231247 (PMC7159244; doi:10.1371/journal.pone.0231247)
Supplement: S1 Table — (DOCX) [file pone.0231247.s003.docx]

**S3 Table. Body mass of sampled gammarids.** Body mass (mean ± se) of gammarids of the four populations and two MOTUs, sampled for measurements of field carotenoid concentrations and immune parameters, and measurements at 15 days and 21 days of diet supplementation with carotenoids in the laboratory. *N* = 30 gammarids per sampling, population, and diet treatment.

| MOTU | Population | Field  collection | Diet supplementation | | | |
| --- | --- | --- | --- | --- | --- | --- |
|  |  |  | 15 days | | 21 days | |
|  |  |  | Control | Supplemented | Control | Supplemented |
| Gf I | Doulonne | 14.4 ± 0.6 | 13.7 ±0.6 | 12.9 ± 0.4 | 13.1 ±0.4 | 13.7 ± 0.5 |
|  | Norges | 11.6 ± 0.4 | 13.5 ± 0.5 | 12.6 ± 0.4 | 13.7 ± 0.6 | 12.3 ± 0.6 |
| Gf VII | Ource | 17.8 ± 1.0 | 16.4 ± 1.1 | 16.8 ± 1.2 | 17.9 ± 0.7 | 17.8 ± 1.2 |
|  | Vivier | 35.6 ± 1.7 | 38.2 ± 2.3 | 31.8 ± 2.2 | 32.8 ± 1.6 | 30.9 ± 2.0 |
