# Supplementary material for: Origin of the natural variation in the storage of dietary carotenoids in freshwater amphipod crustaceans
Source: PLoS One. 2020 Apr 15;15(4):e0231247. doi: 10.1371/journal.pone.0231247 (PMC7159244; doi:10.1371/journal.pone.0231247)
Supplement: S2 Table — (DOCX) [file pone.0231247.s004.docx]

**S4 Table. Mortality of gammarids during diet supplementation**

In addition to the gammarids dedicated to the samplings at day 15 and day 21 during diet supplementation, 40 gammarids per diet treatment were maintained to record the mortality.

**Table S4.** Dead gammarid counts for each diet treatment and each of the four populations, and corresponding values of the χ^2^ test comparing diet treatments for each population. For each test, *df* equals 1, and the significance threshold χ^2^ is 7.82.

| MOTU | Population | Control | Supplemented | Χ^2^ |
| --- | --- | --- | --- | --- |
| Gf I | Doulonne | 11 | 13 | 0.17 |
|  | Norges | 7 | 7 | 0 |
| Gf VII | Ource | 16 | 7 | 3.52 |
|  | Vivier | 20 | 16 | 0.44 |
